# Effectiveness of early interventions for substance-using adolescents: findings from a systematic review and meta-analysis

**DOI:** 10.1186/1747-597X-7-25

**Published:** 2012-06-14

**Authors:** Tara Carney, Bronwyn Myers

**Affiliations:** 1 Alcohol & Drug Abuse Research Unit Medical Research Council, Cape Town, South Africa; 2 Department of Psychiatry and Mental Health, University of Cape Town, Cape Town, South Africa

**Keywords:** Systematic review, Early intervention, Substance use, Behavioural outcomes, Adolescence

## Abstract

**Background:**

Information on the impact of available interventions that address adolescent substance use and delinquency can inform investment choices. This article aims to identify and evaluate early interventions that target adolescent substance use as a primary outcome, and criminal or delinquent behaviours as a secondary outcome.

**Method:**

A systematic review of early interventions for adolescent substance use and behavioural outcomes was conducted.

**Results:**

We identified nine studies using specific search strategies. All but one of the studies reported the use of brief intervention strategies. Only seven studies contained information which allowed for the calculation of an effect size, and were therefore included in the meta-analysis. The overall effect size for all outcomes combined was small but significant (*g* = 0.25, *p* < 0.001). The overall outcome for substance use was also small but significant (*g* = 0.24, *p* < 0.001). For studies with behavioural outcomes, the overall effect size reached significance (*g* = 0.28, *p* < 0.001). In general, subgroup analysis showed that individual interventions with more than one session had a stronger effect on the outcomes of interest.

**Conclusions:**

Early interventions for adolescent substance use do hold benefits for reducing substance use and associated behavioural outcomes. Interventions are most promising if delivered in an individual format and over multiple sessions. One intervention in particular had large effect sizes. As all the interventions were tested in developed countries, further testing is needed in low- and middle-income countries where there is a lack of research on evidence-based interventions for adolescent risk behaviours. Additional recommendations for policy and practice are provided in this paper.

## Background

Adolescence is a critical period for developmental outcomes. Besides experimentation and initiation of substance use that often occurs during this time, previous research shows a relationship between the early onset of substance use and an elevated risk for the later development of substance use disorders [1]. This is concerning as substance use poses a risk for delayed social and academic development, and may impact on brain development among adolescents [2]. In addition, a large body of evidence suggests that substance use is a risk factor for other problem behaviours among adolescents including withdrawal from school involvement, drinking and driving, violent behaviour and general delinquency [3-6]. The longer substance use continues, the more likely it will be associated with harms in various areas, such as health and social problems [7], including involvement in crime. It is therefore important to intervene early with adolescents who use substances and exhibit other problematic behaviours as these behaviours can lead to a number of negative (often long-term) consequences for adolescents.

Studies show that substance use during adolescence plays a role in criminal behaviour in a number of ways. For example, youth offenders in a recent study [8] reported that they committed crimes in order to finance their drug habit. Some participants in this study also reported that using alcohol and drugs gives young people the courage to commit their crimes while others said that substance use sometimes leads to impulsive acts [9] including involvement in criminal activities.

Much research exists on the positive relationship between substance use and delinquent-type behaviours, such as truancy [4], aggression [5,10] and the perpetration of violence through carrying weapons to school or being involved in physical fights [11]. This has fuelled global efforts to reduce adolescent crime through preventing and treating adolescent substance use. While there has been a positive shift towards developing comprehensive policies and services for substance-using adolescents, especially in juvenile-justice populations in high-income countries [12,13], for the most part these risk behaviours have been addressed as two separate issues. Most of the adolescent prevention and treatment efforts have focused on adolescent substance use and have not directly addressed the delinquent-type behaviours that often accompany substance use. This is especially the case in low- and middle-income countries where substance use and crime prevention intervention efforts for adolescents are relatively new.

An example of the sole focus on substance use is a recent systematic review that tested the effectiveness of brief interventions for substance-using adolescents [14] and a similar review [15] that evaluated the effectiveness of motivational interviewing interventions for changing adolescent substance use. While these reviews found small but significant effect sizes, they only measured substance use outcomes. It is possible that the setting in which these interventions occurred influenced the choice of outcome, with most of these interventions taking place in drug treatment settings. In contrast, school-based interventions have been shown to target a broader range of behavioural outcomes, including bullying, substance use, aggression and delinquent behaviour [16].

Only a small number of reviews [14,15] investigated the effectiveness of these substance use interventions. This is partly because the clinical need for adolescent-oriented substance use services has outweighed the need to evaluate the effectiveness of these services [17]. Yet it is crucial for research to address which kind of intervention and mode of delivery is effective for high-risk adolescents so that investments can be made in safe and appropriate services. For example, peer-group interventions have been shown to be harmful in some instances, leading to increases in problem behaviours and contributing to negative life outcomes during adulthood [18], yet these remain popular in some contexts. With changes in the global economic climate there has been increasing pressure on service planners and policy makers to show that the interventions they are proposing are evidence-based and cost-effective. Findings from systematic reviews of interventions are useful in this regard as they can guide choices about which interventions are the best to invest in. This is especially important in resource-strapped settings.

This article aims to redress this lack of evidence on the effectiveness of interventions to address adolescent substance use and associated behavioural problems through conducting a systematic review of early interventions targeting these dual outcomes. More specifically, the primary objective of this review was to summarize the evidence, and assess the effectiveness of early-interventions for substance-using adolescents at risk for delinquency and involvement in crime. In this review, early interventions are defined as interventions for adolescents who are already using substances but who do not meet diagnostic criteria for substance abuse or dependence and therefore may not need specialist drug treatment services [19].

## Methods

### Inclusion and exclusion criteria

To be included in the review, studies had to meet several criteria. First, studies had to compare an early intervention to treatment or care as usual. In this review we defined an intervention as an early intervention if it targeted adolescents who did not meet criteria for abuse or dependence but were already using alcohol or other drugs and had a screening component for alcohol and other drug use [20] as well as an intervention component. These early interventions could also include brief interventions, which have less of an emphasis on advice-giving by the interventionist than other types of interventions [21].

Second, studies were only included in the review if participants were assigned to an experimental and control group. Studies that did not have any comparison group of subjects, and also qualitative studies, were excluded from the review. Third, the studies were also required to have pre-test and post-test or follow-up measures. Fourth, for a study to be included in the review, the intervention had to have behavioural outcomes in addition to substance use. Specifically, the intervention had to have changes in alcohol or other drug use and problem behaviours (related to delinquent or criminal behaviour) as its primary and secondary outcomes respectively. The use of alcohol and other substances with the potential to be abused (including heroin, cocaine, cannabis, methamphetamine, methaqualone, over-the-counter and prescription medicines) were included as primary outcomes. Secondary outcomes included delinquent-type behaviours that could be legal (such as truancy from school, aggression and fighting) as well as behaviour that could have legal consequences (such as shoplifting or theft, assault, damage to property). Studies that only had attitudinal change as a main outcome were excluded from the review.

Studies that focused on tobacco use *only* were excluded from this study, as previous systematic reviews have provided very limited evidence for the effectiveness of interventions that focus only on changing tobacco use among adolescents [22]. Other inclusion criteria were age-related, with participants having to be between 13 and 19 years of age, as the focus of this review was on interventions for adolescents.

### Search strategy to identify studies

**Figure 1:**
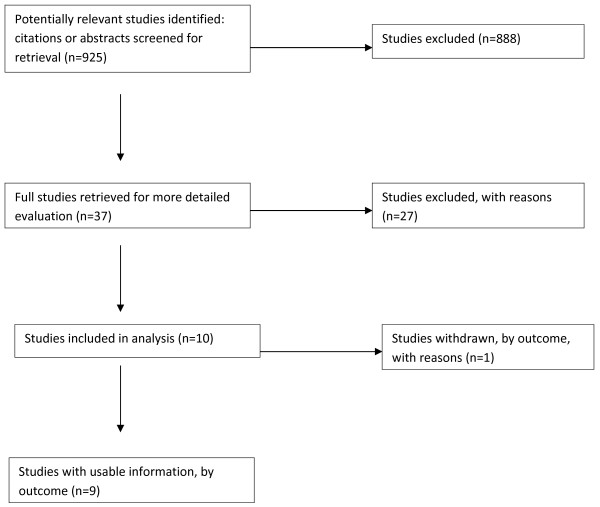
The selection process for studies included in the systematic review.

Searches were developed and then run on the following electronic databases: Embase, ERIC, Cochrane Database of Systematic Reviews, ISI Web of Knowledge Social Science Citations, LILACS (Latin American and Caribbean Health Sciences), ETOH, PsychINFO, PubMED and ISAP. We also searched the US Substance Abuse Mental Health Services Administration's (SAMHSA) repository for evidence based programmes on substance use, with crime/delinquency and violence as secondary outcomes. The basic search terms used were: (adolescent/adolescence or teenager), and (early or brief or minimal intervention or counselling) and (drugs: cannabis, cocaine, heroin, amphetamine, prescription, methaqualone and alcohol) and (drinking behaviour or binge drinking) and (substance use or abuse or misuse). In addition, the main author manually searched through the reference lists of the selected studies to see if any of the references referred to studies which potentially met the inclusion criteria. The author also did not only limit the search to English language publications. Nine-hundred and twenty-five abstracts were found through conducting this search. Two independent authors then looked at the selected abstracts that appeared to be potentially relevant (based on above-mentioned inclusion criteria). They met to discuss any differences in their selections, but it was unnecessary to bring in a third author to resolve any of these differences. Thirty-seven full-text articles (including two Spanish articles which were translated) were then obtained and the same two authors read these and completed a table to decide whether all inclusion criteria were met. Based on the inclusion criteria, ten articles were chosen for analysis, but one article did not contain clear outcomes and therefore was excluded at a later stage (see Figure 1 for selection process).

### Data analysis

Two studies were excluded from the meta-analysis because none of the appropriate results were significant, and were therefore not provided in the results section of the respective articles or available from authors. This left seven studies that contained enough information and had comparable outcomes to allow them to be included in a meta-analysis, where effect sizes (Hedge’s *g*) and standard errors of experimental and control groups were compared. This effect size was chosen because it corrects for biases due to small sample sizes, which was the case for some of the included studies [23]. Hedge’s g can be interpreted in a similar manner to Cohen’s suggested interpretation of effect sizes, whereby 0.20 is considered small, 0.50 is considered medium, and 0.80 is considered large. An effect size calculator allowed the author to calculate effect sizes for various different reported statistics in an attempt to measure overall effect sizes [24].

Calculated effect sizes that were shown to be positive reflected a reduction in risk behaviour (namely, substance use or delinquent-type behaviours) in the direction of the experimental group, indicating that the intervention had a significant effect on decreasing high-risk behaviours. Effect sizes were calculated between the control group and the most intensive experimental group or the experimental group that differed most from the control group (in the event that more than one intervention was included in the study). We analysed only post-intervention outcomes. When studies had more than one follow-up appointment after the intervention, we took an average of the scores across follow-up times by calculating the arithmetic mean, similar to a previous systematic review on alcohol and drug use [14]. The majority of studies reported on a number of outcomes and average effect sizes which were calculated using specialised software (Review Manager) that generated weighted effect sizes [25]. A random effects model was chosen for the meta-analysis. Furthermore, we decided to conduct sub-group analyses for the delivery of intervention (group versus individual), and the length of the intervention (single versus more than one session, including booster sessions) in case of high levels of heterogeneity across the studies, and to make the results easier to interpret.

## Results

### Description of included studies

We identified nine studies with a total of 1895 adolescents (see Table 1 for characteristics) who participated in the studies and 1638 (86%) who participated until completion (namely, they received the full intervention and attended all planned follow-up appointments). The majority of the study participants were male (63.1%). The studies were conducted in different types of education settings: two were conducted in high schools: one public urban high school [26] and one alternative high school [27], and two were conducted in juvenile facilities [28,29]. Other settings included emergency room departments [30,31] community health centres [32,33] and youth centres [34]. Eight of the interventions were brief motivational interventions. The remaining study used an intervention that was of longer duration (55 sessions) and also utilised a three-prong approach [28] which consisted of a life skills training (LST) model (Botvin LST model), the Prothrow/Stith anti-violence model, and an approach that focused on values clarification. One of these interventions was administered in a group [32] while all the other studies used individual-based interventions. Only one of the studies included a secondary population, namely the substance-using adolescents’ parents [26].

**Table 1:** Characteristics of included studies

**Study ID**	**Setting and Country**	**Type of Intervention**	**Gender breakdown (%)**	**Age (Mean, SD)**
1. Grenard et al., 2007 [27]	Alternative high school campuses, Los Angeles, USA	Individual Motivational Interviewing (1 session of 25 minutes)	67% Male, 33% Female	16.1 (0.9)
2. Friedman et al., 2002 [28]	Residential Facility for court adjudicated adolescent males, Philadelphia, USA	Botvin Life Skills Training (20 sessions), Prothrow/Stith Anti-Violence model (20 sessions), Values Clarification procedure-20 sessions: (55 minutes each)	100% Male	15.5 (1.1)
3. Stein et al., 2006 [29]	Northwest juvenile correctional facility, USA	Motivational Interviewing (60 minute , 90 minute booster)	89.5% Male, 10.5% Female	17.09 (1.06)
4. Bailey et al., 2004 [33]	Youth Centre, New South Wales, Australia	Brief Motivational Interviewing group intervention (4 sessions-first session 40 minutes, remaining sessions 30 minutes)	50% Male, 50% Female	15.44 (1.80)
5. Peterson et al., 2006 [34]	Homeless adolescents-drop in centres, street intercepts. Seattle, Washington, USA	Brief Motivational Enhancement (1 session of approximately 30 minutes)	54.7% Male, 45.3 Female	17.4 (1.54)
6. Winters et al., in press [26]	Urban Public High School, Minnesota, USA	Teen Intervene-Brief Motivational Interviewing (2 sessions with adolescent of 60 minutes, 1 session of 60 minutes with parent)	51.5% Male, 48.5% Female	16.1 (n/a)
7. Walton et al., 2010 [31]	Emergency Department, Michigan, USA	SafERteens therapist vs computer brief intervention (1 session of 35 minutes)	43.5% Male, 56.5% Female	16.8 (1.3)
8. Spirito et al. , 2004 [30]	Northeast Emergency Department, USA	Brief Motivational Interviewing (1 session of 35–45 minutes)	63.8% Male, 36.2% Female	15.6 (1.2)
9. D’Amico et al., 2008 [32]	Community-based health clinic, Los Angeles, USA	Project CHAT: Motivational Interviewing (1 session of 15–20 minutes; 5–10 minute booster telephone call	47.6% Male, 52.4% Female	16.0 (1.85)

### Quality of included studies

Overall, the studies were of a high quality, with the majority of them controlling for attrition or low levels of study dropout. The participants in all nine studies were randomly selected to experimental or control groups. Six of these studies were randomized controlled trials [26,30-34], and the remaining studies had quasi-experimental designs [27-29]. Attrition rates varied across the studies. Only one study [32] did not discuss participant withdrawal, and it seems that although there was no drop out, it had a small sample size (total N = 34). Another study [26] also reported a very low attrition rate (1.3%) and therefore did not conduct an intention to treat analysis to control for the effects of attrition. Two of the studies used an intention to treat analysis [31,34]. Others studies found that there were no statistically significant differences between those who withdrew from the study and those that completed the study [30,33]. At baseline, one of the studies [32] found that there were no statistically significant differences between groups, but the results indicated that the trend was for the intervention group to be less likely to complete the six month follow up appointment.

All of the studies included follow-up periods to measure substance use and delinquent behaviour after the administration of interventions, ranging from one to 12 month follow-up periods. Outcomes that were measured and reported on included frequency of alcohol and other drug (AOD) use, quantity of AOD use, and binge drinking or heavy drinking. The behavioural outcomes of substance use included negative consequences of AOD use (including legal problems), aggression and violence and driving under the influence of AODs (such as cannabis) (see Table 2).

**Table 2:** Outcome measures of included studies

**Study ID**	**Outcome**	**FU Group sizes /attrition rate**	Intervention Results: Mean (SD)*	**Control Results: Mean (SD)**	**Hedge’g and SE for Meta-Analysis**
1. Grenard et al,, 2007 [27]	Frequency alcohol Frequency marijuana Frequency other drugs Binge drinking Quantity drinks RAPI	11 Experimental 7 Control 18% attrition	Not significant Not significant Not significant Not significant Not significant Not significant	Not significant Not significant Not significant Not significant Not significant Not significant	Unable to calculate
2. Friedman et al., 2002 [28]	Frequency drug use Frequency alcohol Frequency illegal offenses Drug Selling Violent offences School problems	110 Experimental 91 Control 16% attrition	6.4 3.1 49.9 17.7	n/a	Only for experimental group
3. Stein et al., 2006 [29]	Frequency of drinking and driving Frequency of marijuana use and driving Frequency of driving with driver who had been drinking Frequency of driving with driver who had used marijuana	59 Experimental, 45 Control	0.43 (1.3) 6.25 (15.78) 4.68 (11.38) 19.07 (30.59)	2.32 (4.18), 11.09 (21.4) 11.16 (13.85) 23.77 (30.94)	0.64 (0.20) 0.26 (0.13) 0.52 (0.20) 0.15 ( 0.08)
4. Bailey et al., 2004 [33]	Frequency of alcohol Quantity alcohol Binge drinking Hazardous drinking Risk-taking behaviors	Experimental: 17, Control: 17	1.49 (0.86), 1.72 (1.37), 1.57 (0.7), 4.79 (2.46), 2.39 (1.39)	1.63 (0.98), 1.67 (1.28), 1.67 (1.03), 4.96 (2.83), 1.71 (1.21)	0.15(0.08), 0.04(0.02), 0.11(0.05), 0.07(0.04), 0.50(0.27)
5. Peterson et al., 2006 [34]	Binge drinking Frequency alcohol use Quantity alcohol Frequency marijuana Frequency days of illicit drug use summed days of illicit drug use RAPI: drug use consequences	212 (69, 77, 65) (20% attrition) Intention to treat	Not significant Not significant 12.72 (11.54) 7.89 (10.32) 8.99 (13.05) Not significant	Not significant Not significant 12.39 (12.7) 7.69 (8.87) 10.63 (15.61) Not significant	0.06 (0.03) 0.07(0.04) 0.15(0.08)
6. Winters et al., in press [26]	Frequency alcohol Frequency marijuana # alcohol abuse symptoms #alcohol dependence symptoms # cannabis abuse symptoms #cannabis dependence symptoms PCS	Experimental 1(adolescent): 134, Experimental 2(parent and adolescent): 121, Control: 55 Attrition rate: 1.3%	2.8 (4.4), 8.3 (14.3), 0.4 (1.1),0.7 (1.6), 4.3 (3.4), 1.0 (2.1), 12.1 (2.0)	10.5 (11.8), 14.9 (18.1), 1.3 (1.9), 2.6 (3.9), 1.8 (2.6), 2.2 (3.0), 13.5 (3.1)	1.01 (0.08), 0.42 (0.16), 0.64 (0.17), 0.75(0.17), 0.79(0.17), 0.50(0.16), 0.58(0.17)
7. Walton et al., 2010 [31]	Alcohol use frequency/ Quantity of alcohol use Binge drinking Alcohol consequences Peer Aggression Violence	Experimental 1(Comp): 209, Experimental 2(Therapist: 209, Control: 208 Attrition rate: 15% Intention to Treat	3MFU: 0.25 (0.22), 6MFU: 0.19 (0.23) 3MFU: 0.03 (0.22), 6MFU: 0.02 (0.23) 3MFU: 0.41 (0.23), 6MFU: 0.59 (0.25) 3MFU: 0.30 (0.10); 6MFU: 0.17 (0.11) 3MFU: 0.41(0.23); 6MFU: 0.08 (0.09)		0.22(0.23), 0.03(0.23), 0.05(0.24), 0.24(0.11), 0.25(0.16)
8. Spirito et al., 2004 [30]	Frequency alcohol Quantity alcohol High/binge drinking drinking and driving	Experimental: 64, Control: 60 Attrition rate: 19%	3.07 (4.25), 3.19 (2.56), 1.59 (2.97) Not significant	4.15 (5.66), 3.36 (2.95), 2.58 (4.33) Not significant	0.21(0.11), 0.06(0.03), 0.27(0.14)
9. D’Amico, Miles, Stern & Meredith, 2008 [32]	Alcohol consequences Frequency alcohol Quantity of drinks Heavy drinking (+3 drinks) Marijuana	Experimental: 22, Control: 20 Attrition: 34%	n/a	n/a	0.07 (0.14), 0.42 (0.63), 0.11 (0.42), 0.20 (0.35), 0.30 (0.19), 0.77 (0.26), 0.44 (0.56)

*Note: for Walton et al. (2010) and Damico, Miles, Stern & Meredith (2008) the results were already provided as effect sizes and standard errors.

### Intervention effects

**Figure 2:**
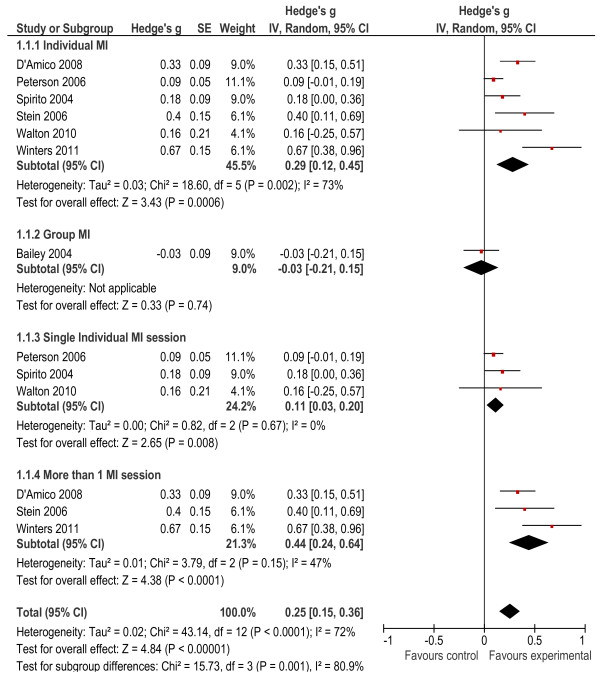
Forest plot of all outcomes.

The weighted overall effect size was calculated from seven studies. Two studies [27,28] did not include enough information on the results to calculate all appropriate statistics and were, therefore, excluded from the meta-analysis. The overall effect size was calculated as *g* = 0.25 (*p* < 0.001), and significantly favoured the direction of the intervention (experimental group). The results of the subgroup analysis indicated that there was a difference in delivering the intervention in a group setting (*g* = −0.03, *p* = 0.74) [33] in comparison to an individual setting (*g* = 0.29, *p* < 0.001) showing better outcomes for interventions delivered in individual formats (Figure 2). When overall effect sizes were considered for individual studies, six of the seven interventions effect sizes were small [29-34], while only one intervention showed a medium to large effect [26]. The meta-analysis results did, however, indicate a large degree of heterogeneity between the study outcomes (*X*² = 43.14, *p* < 0.001), with I^2^ (72%) indicating the percentage of the variability in effect estimates that is due to heterogeneity. We therefore conducted further analyses that took into the account the number of intervention sessions provided, by performing subgroup analysis by grouping studies together with similar characteristics. Results indicated that both single (*g* = 0.11, *p* = 0.008) and multiple intervention sessions (*g* = 0.44, *p* = <0.001) had a significant effect on the outcomes, but the effect size was larger for multiple-session interventions. Levels of heterogeneity were not significant for either type of intervention.

#### Comparison of primary outcomes

**Figure 3:**
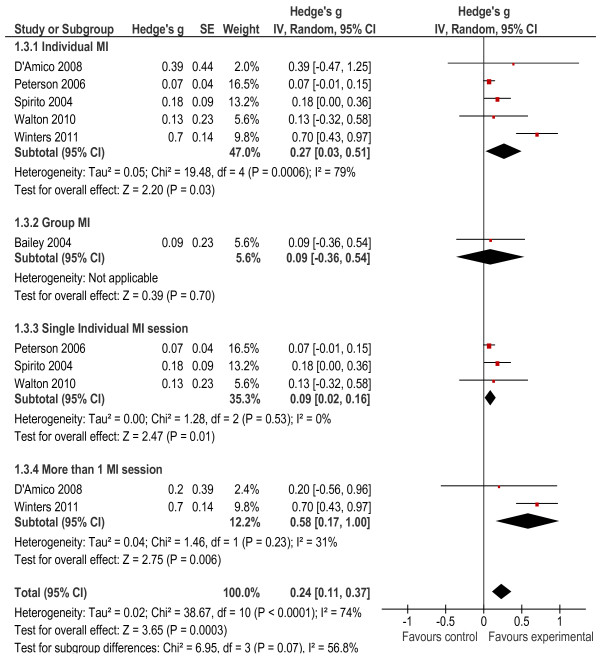
Alcohol and other drug outcomes.

We first compared the AOD outcomes across six of the seven studies, as one only measured risky behavioural outcomes associated with substance use [29]. These outcomes included frequency of use, quantity of use, heavy alcohol use and symptoms of misuse and abuse (see Figure 3). The meta-analysis results showed that when alcohol and drug outcomes were considered together, the overall effect size was significant at *g* = 0.24 (*p* < 0.0001). The largest effect size for the AOD outcomes was 0.70 [26]. The heterogeneity statistics also indicated that the heterogeneity between studies was significant (*X*^2^ = 38.67, *p* < 0.001, I^2^ = 74%). Once a subgroup analysis was conducted, individual interventions had a significant effect (*g* = 0.27, *p* = 0.03) while the group-delivered intervention did not have a significant effect (*g* = 0.09, *p* = 0.70). The heterogeneity for single-session and multiple-session interventions was not significant. Having more than one session seemed to have a bigger effect (*g* = 0.58, *p* = 0.006) on AOD outcomes than a single intervention session (*g* = 0.09, *p* = 0.01).

##### *Alcohol frequency:*

**Figure 4:**
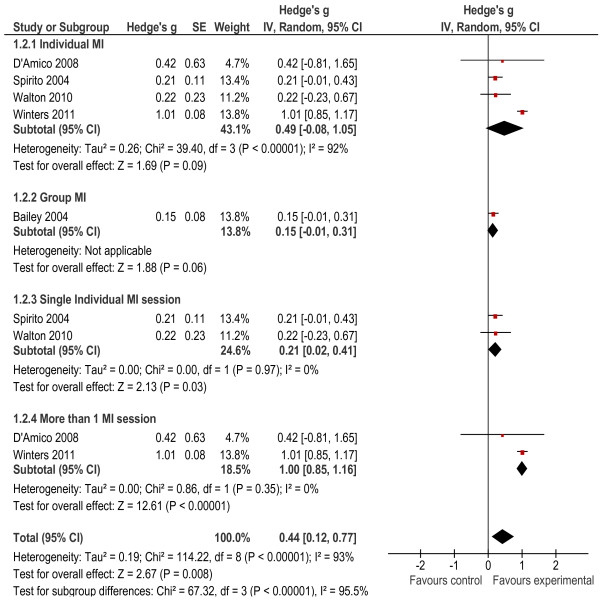
Alcohol Frequency Outcomes.

Five of the seven studies measured alcohol frequency [26,30-33] (defined as the number of times alcohol was used in the 30 days prior to the interview). The findings for one study were excluded because they were not provided [34]. The overall effect size was significant at 0.44 (*p* = 0.008). Also, the heterogeneity statistics indicated that the heterogeneity between studies was significant (*X*² = 114.22, *p* < 0.001, I^2^ = 93%). The subgroup analysis results indicated that the intervention delivered in a group format did not have a significant effect on alcohol frequency (*g* = 0.15, *p* = 0.06), nor did the intervention delivered in an individual format significantly reduce alcohol frequency (*g* = 0.49, *p* = 0.09). Further subgroup analysis showed that the provision of both single (*g* = 0.21, *p* = 0.003) and multiple session individual interventions (*g* = 1.00, *p* < 0.001) was effective, although the intervention effect was greater when delivered across multiple sessions. We found acceptable levels of heterogeneity for these interventions (see Figure 4).

##### *Alcohol quantity:*

**Figure 5:**
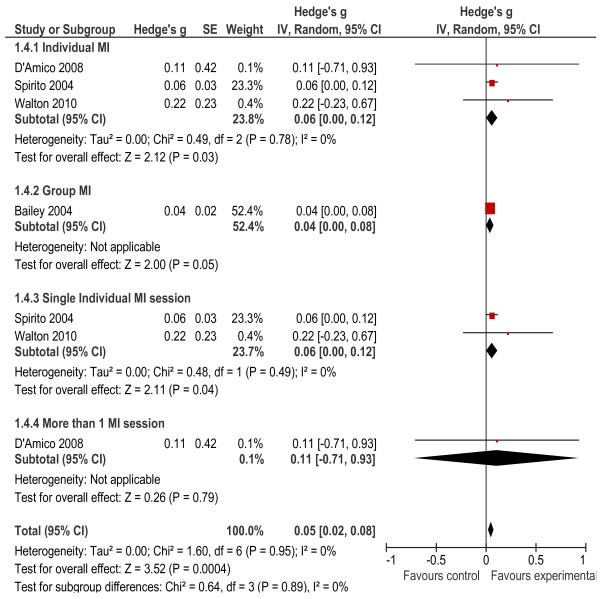
Alcohol quantity outcomes.

Four of the studies [30-33] measured alcohol quantity (how many drinks participants consumed on a typical drinking day 30 days prior to the interview). The effect size was small at 0.05, but significant (*p* < 0.001). There was no significant heterogeneity between studies (*X*² = 1.60, *p* = 0.95, I^2^ = 0%). Subgroup analyses showed that interventions delivered on an individual basis had a significant effect (*g* = 0.06, *p* = 0.03), as did the intervention delivered in a group setting (*g* = 0.04, *p* = 0.05). Studies that delivered a single individual session had a significant effect on alcohol quantity (*g* = 0.06, *p* = 0.04), while the study that delivered more than one session did not have a significant effect (*g* = 0.11, *p* = 0.79; Figure 5).

##### *Binge drinking*

**Figure 6:**
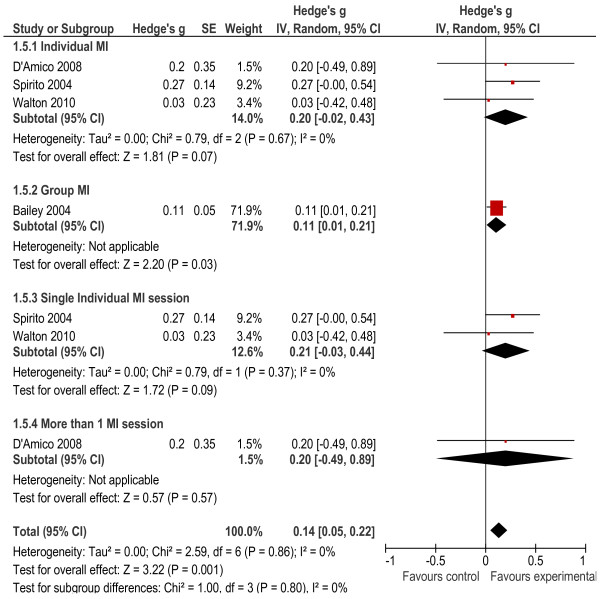
Heavy/Binge drinking.

Four studies [29,31-33] measured binge drinking as drinking three, five, six or more drinks respectively at one time period among adolescent participants. The overall mean difference score was significantly different to zero (*g* = 0.14, *p* = 0.001). No significant heterogeneity was found between studies (*X*² = 2.59, *p* = 0.86, *I²* = 0). The interventions delivered to individual adolescents did not significantly reduce binge drinking (*g* = 0.20, *p* = 0.07) while the one delivered in a group format did have a significant effect on binge drinking (*g* = 0.11, *p* = 0.03). Subgroup analysis regarding the mode of intervention delivery (individual vs. multiple sessions) did not yield any significant differences (see Figure 6).

##### *Marijuana/Cannabis frequency:*

**Figure 7:**
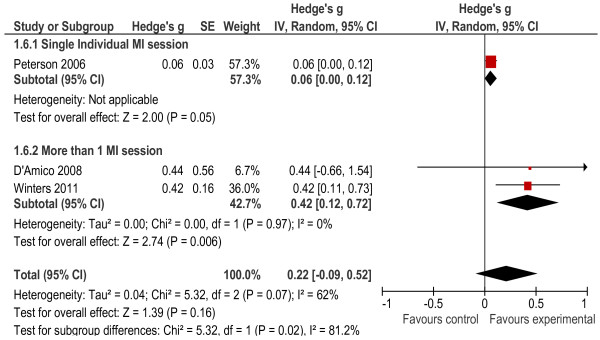
Marijuana use outcomes.

Three of the studies contained a measure that asked study participants about how often they used marijuana in the 30 days prior to the interview [26,32,34]. The overall effect size was 0.22 and was not significant (*p* = 0.16). The heterogeneity statistics also indicated that the heterogeneity between studies was acceptable (*X*² = 5.32, *p* = 0.07, I^2^ = 62%). All of the studies delivered individual interventions. While single-session interventions had a significant effect on outcomes (*g* = 0.06, *p* = 0.05), a stronger effect size was obtained for multiple-session interventions (*g* = 0.42, *p* = 0.006) which had acceptable levels of heterogeneity (see Figure 7).

#### Comparison of secondary outcomes

**Figure 8:**
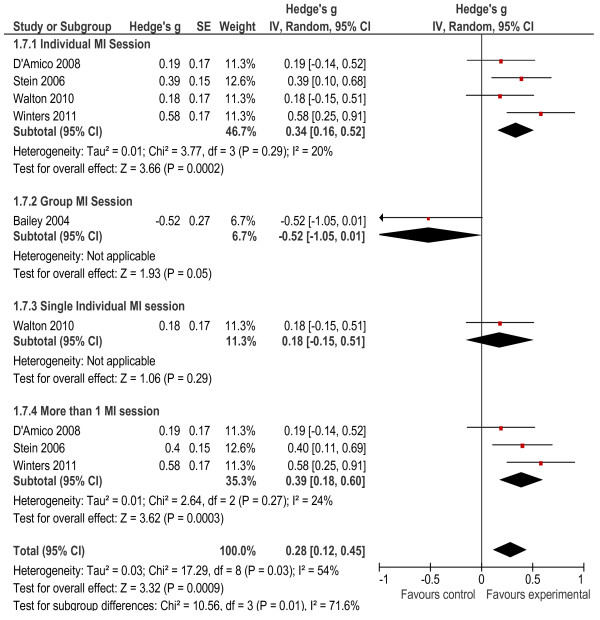
Behavioral outcomes.

**Figure 9:**
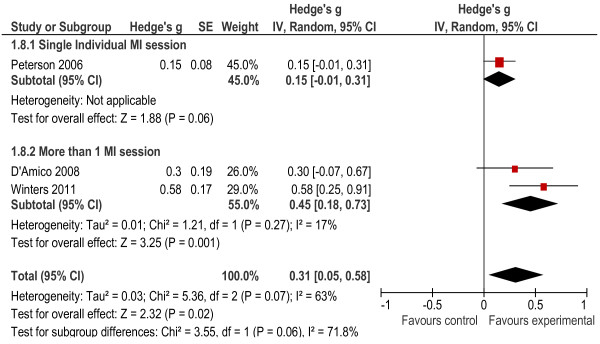
Drug use consequences.

**Figure 10:**
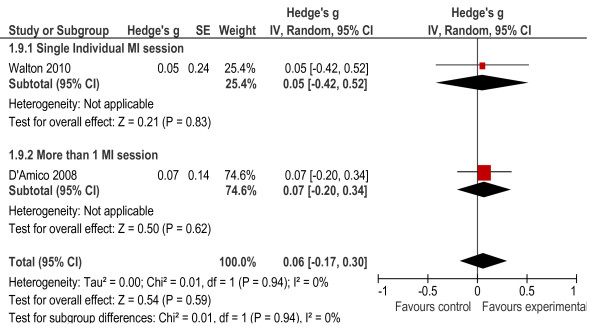
Alcohol consequences.

A number of behavioural outcomes were measured in the seven studies included in the meta-analysis. These included specific instruments that measured risky behaviours associated with AOD use, such as the Rutgers Alcohol Problem Index (RAPI), which examines AOD-related fighting and aggressive behaviour [34] and the Personal Consequences Scale (PCS) which examines legal consequences and other problematic behaviours [26]. Other outcomes included driving under the influence of AODs [29,30], violent and aggressive behaviours [31], and other risky behaviours [33] and consequences [26,31,32,34] associated with AOD use. Two of the seven studies were excluded because findings on behavioural outcomes were not provided [30,34]. The results of the meta-analysis on behavioural outcomes reached statistical significance (*g* = 0.28, *p* < 0.001). In addition, the heterogeneity statistics indicated that the heterogeneity between studies was significant (*X*² = 17.29, *p* = 0.03, I^2^ = 54%) and it was therefore difficult to compare findings across studies (see Figure 8). To further understand findings, subgroup analyses were conducted. These showed that interventions delivered in an individual format (*g* = 0.34, *p* < 0.001) had a significant effect on behavioural outcomes, while the control group showed better outcomes than the experimental group for the intervention delivered in a group format (*g* = −0.52, *p* = 0.05). Individual interventions that consisted of multiple sessions (*g* = 0.39, *p* < 0.001) had a significant effect on behaviour outcomes, while those that consisted of single sessions did not have a significant effect (*g* = 0.18, *p* = 0.29). Heterogeneity levels were acceptable for these subgroup analyses. Further analysis showed drug use consequences were significant (*g* = 0.31, *p* = 0.02) but alcohol consequences (*g* = 0.06, *p* = 0.59) were not significant (see Figures 9 and 10). A subgroup analysis showed that the provision of more than one intervention session significantly reduced drug use consequences (*g* = 0.45, *p* = 0.001).

## Discussion

This review is the first of its kind to examine the impact of early interventions on adolescent substance use and behavioural outcomes. To the best of our knowledge, there are no other reviews that measure the effect that brief interventions have on substance use *and* behavioural outcomes. This review therefore adds valuable evidence for interventions that address two risk behaviours simultaneously. This is important for policy makers for a number of reasons. First, all the interventions included in the meta-analysis reported significant reductions in drug and alcohol use, providing strong evidence in support of the effectiveness of early interventions for adolescent substance use. This indicates the effectiveness of treating adolescents early as their substance use progresses, that is, before they need specialised treatment [19] or face unwanted consequences such as incarceration.

Second, the majority of the early interventions included in this systematic review were brief in nature. This suggests that interventions do not need to be lengthy to be effective with adolescents. This is important for policy makers as there are cost implications associated with lengthier interventions. Brief interventions are among some of the most cost-effective types of behavioural treatments [35], and may be especially useful in low- and middle-income countries where health care systems are burdened and have few resources available for intensive behavioural interventions. To ensure that adolescents are reached early, they should be targeted while still at school. Furthermore, screening for identification of appropriate adolescents should be conducted in a number of places. This could possibly further take some of the burden off more specialised treatment providers, and provide services to adolescents who do not have access to the health care system [19].

Third, this review provides evidence for the format best suited to delivering these interventions to at-risk adolescents. When subgroup analyses were conducted to compare the effectiveness of interventions delivered in individual versus group format and those delivered over single versus multiple intervention sessions, a clear pattern of findings emerged which favoured the delivery of interventions in an individual-format and over multiple sessions. For example, interventions delivered in an individual format across multiple sessions had a much greater effect on the frequency of alcohol use and the frequency of cannabis use than single-session interventions or those delivered in a group-format.

Similarly, the results of the overall meta-analysis found that the interventions had a significant effect on problem and criminal behaviours related to substance use, and subgroup analyses found that interventions delivered in an individual-format and over multiple sessions had significant, albeit relatively small, effects on behavioural outcomes. In contrast, interventions delivered in group formats or in single sessions did not have the desired effect on behavioural outcomes [33]. This is in keeping with findings from a previous study [18] that found group interventions to be harmful for high-risk adolescents, for both short- and longer-term outcomes such as convictions for criminal activities and psychiatric problems. Further, a single intervention session may not be long enough to change relatively entrenched behaviours. While these findings of the subgroup analysis suggest that effective early interventions for addressing adolescent substance use and related problem behaviours should be in an individual-format and delivered over multiple sessions, this needs to be further researched as the effect sizes (particularly for behavioural outcomes) remain small and few studies have directly examined behavioural outcomes for substance use interventions delivered in group formats. In low- and middle-income countries especially, where resources are often limited, group-format interventions are cost effective and providing a one-on-one intervention in schools may not always be feasible or affordable. Furthermore, there was only one group-format intervention that met the inclusion criteria for this review, which cannot be generalised to all types of group interventions. Clearly this is an area that warrants more research in low- and middle-income countries before policy recommendations can be made.

It should be noted that the small effect sizes obtained could be due to several factors, including measurement issues. For instance, some studies used measures that included multiple consequences of substance use that were not necessarily related to the behavioural outcomes of interest, and these could not be separated out in the meta-analysis (for example, getting in to trouble with family [32]). Also, the time frames of the follow-up measurements may have influenced the results. Brief interventions can have immediate effects on substance use outcomes but other behavioural outcomes are often more distal outcomes of these interventions. It may be necessary to conduct research that includes longer follow up periods to accurately assess whether the intervention has an impact on other behavioural outcomes.

Fourth, the review is useful as it provides clear guidelines regarding the strength of interventions and also points to interventions that seem more likely to be effective than others. This will be of value to policy makers who are looking for a strong evidence base to guide the adoption of one intervention vis-à-vis others. Furthermore, some of the studies clearly seemed more effective than others, for example the outcome effect size for Winter et al’s [26]*Teen Intervene* were generally consistently larger than that of the other studies. *Teen Intervene* has been registered with the U.S. National Registry of Evidence-based Programs and Practices since 2007 [36]. Of the nine interventions included in this systematic review, it is the only intervention that included a session with the adolescents’ parents. Previous research has indicated that the involvement of key family members in interventions with substance-using adolescents who also face other problems (such as truancy and involvement in justice-diversion programs) is useful and may lead to sustained outcomes [37]. This review clearly suggests that *Teen Intervene* is a powerful intervention for adolescents who use substances. However, it may need to be expanded to include a stronger focus on delinquent-type behaviours (and not just legal consequences associated with substance use).

Findings from this study should be considered in the light of some limitations. First, there was substantial heterogeneity between the studies. In this systematic review, the length of follow up appointments and study quality, especially in defining and reporting outcomes, varied substantially across studies. This highlights the need for standardised outcome measures for substance use and other behavioural outcomes, as well as guidelines for the choice of follow up periods for intervention studies. Another weakness was that only two of the studies reported an intention to treat analysis, and while one of the remaining studies had an extremely low loss to follow-up rate [26], the remaining results could have been somewhat biased if participant follow-up was related to their response to treatment.

A final limitation is that all the included studies are from developed country settings (primarily the USA or UK) and therefore it is unclear whether these findings can be generalised to low- and middle-income countries. Although there is some promising intervention work on substance use and other problem behaviours from developing regions, none of these studies met the inclusion criteria for this review as they were mainly descriptive, non-experimental studies [38]. This highlights the need for more intervention research in developing regions that address the interlinked risks of substance use and problem behaviours. A recommendation for further work is, therefore, to test the cultural applicability of the recommended intervention from this review *(Teen Intervene)* to a developing country setting such as South Africa.

Third, as we did not expect to find a large number of intervention studies that addressed substance use and behavioural outcomes, the inclusion and exclusion criteria for this review were not as rigorous as they could have been as studies did not necessarily have to be randomized controlled trials. Future systematic reviews that are not as exploratory in nature should aim to include only randomized controlled trials in so that stronger evidence of intervention effectiveness can be provided.

Finally, results were difficult to compare across studies for two reasons: (a) few studies were found that examined behavioural outcomes directly and; (b) the five studies that included these outcomes used different measurement tools. This clearly highlights the need for future studies that provide integrated interventions that directly target substance use as well as substance-related behavioural outcomes within the context of the intervention.

## Conclusions

The findings for this systematic review clearly demonstrate the value of early interventions for effectively targeting adolescent substance use and that these can reduce substance use and also impact on other behavioural outcomes. Interventions that are delivered in an individual format and across multiple-sessions seem particularly beneficial. This is important as it provides rationale for substance use intervention programs that measure additional outcomes to alcohol and drug use. Although the impact on behavioural outcomes was small, it was significant, necessitating the need to improve measurement of behavioural outcomes in future studies and to also have a stronger focus on this material in future interventions that hope to reduce substance-related problem behaviours. Finally, one study consistently had larger effect sizes than any of the other interventions [26]. As this promising intervention has not been tested in a developing country context, a recommendation from this systematic review is to test this intervention in a low- and middle-income country, following cross-cultural adaptations. This would greatly add to the body of research on evidence-based interventions for this subpopulation of at-risk adolescents.

## Competing interests

The authors declare that they have no competing interests.

## Authors’ contributions

TC conducted the search for articles, drew up the data extraction form, selected relevant studies, analysed the data and contributed to writing the manuscript; BM contributed to the selection of relevant studies and writing of the manuscript. Both authors read and approved the final manuscript.
